# Effect of metformin and detorsion treatment on serum anti-Müllerian hormonelevels and ovarian histopathology in a rat ovarian torsion model

**DOI:** 10.3906/sag-1803-196

**Published:** 2020-04-09

**Authors:** Sema KARAKAŞ, Cihan KAYA, Hakan GÜRASLAN, Damlanur SAKIZ, Sema SÜZEN ÇAYPINAR, Hüseyin CENGİZ, Murat EKİN, Levent YAŞAR

**Affiliations:** 1 Department of Obstetrics and Gynecology, University of Health Sciences,Gaziosmanpaşa Taksim Training and Research Hospital, İstanbul Turkey; 2 Department of Obstetrics and Gynecology, University of Health Sciences,Bakirköy Dr. Sadi Konuk Training and Research Hospital, İstanbul Turkey; 3 Department of Pathology, University of Health Sciences,Bakirköy Dr Sadi Konuk Training and Research Hospital, İstanbul Turkey

**Keywords:** anti-Müllerian hormone, detorsion, metformin, ovarian reserve, ovarian torsion

## Abstract

**Background/aim:**

Adnexal torsion is a common gynaecological emergency, and considered to be a problem mostly in reproductive-age women. To evaluate the effect of metformin and detorsion treatment on reducing ovarian reserve in an ovarian torsion model.

**Materials and methods:**

Twenty-four nonpregnant, Wistar Hannover rats were included in the study. Animals were divided into 3 groups: the control group, the detorsion only group, and the metformin + detorsion group. The first group received only laparotomy. In the second group, ovaries were fixed to the abdominal wall after performing 360° ovarian torsion, followed by detorsion after a 3-h period of ischemia. The third group underwent the same torsion and detorsion procedures as the second group, and received 50 mg/kg metformin by gavage for 14 days. Ovarian damage scores, follicle counts, and AMH levels were evaluated.

**Results:**

The total damage score was significantly increased in the detorsion only group compared to the metformin+detorsion and control groups. Pre-operative/post-operative AMH decreases were statistically significant in negative direction in the detorsion only group when compared to the metformin+detorsion and control groups (P = 0.001).

**Conclusion:**

Metformin+detorsion treatment may be effective in protecting the ovarian reserve after ovarian torsion.

## 1. Introduction

Ovarian torsion is defined as partial or complete rotation of the ovary around its pedicle or vascular axis [1,2]. Torsion of adnexal structures can result in massive parenchymal congestion, infarcts, and haemorrhagic necrosis after arterial and venous blockade [2]. Adnexal torsion is the fifth most common gynaecological emergency, with a reported incidence of 2.7% in the United States [3,4]. Although ovarian torsion is considered to be a problem mostly in reproductive-aged women, it can occur from early foetal life to the postmenopausal period [5]. 

Owing to its nonspecific symptoms, such as nausea, vomiting, and pelvic pain, there is almost always a delay in diagnosis. Ultrasound imaging may be helpful in the diagnosis. However, even Doppler sonography is successful in diagnosing only 40% of surgically confirmed cases [6]. This difficulty in diagnosing ovarian torsion leads to loss of ovarian tissue and function. A total of 50%–90% of adnexal torsion cases are caused by physiological cysts, endometriosis, dermoid cysts, fibromas, and other benign or malignant ovarian neoplasms [7,8]. Traditionally, the suggested treatment is salphingoophorectomy or oophorectomy. However, evaluating tissue perfusion intraoperatively and leaving the tissue in its anatomical place is a common and reliable approach, especially for reproductive-aged women [9,10]. There is no relation between the variable colours (purple to black) of the adnexal structures and tissue viability.

It has also been reported that detorsion of the adnexa has no effect on the risk of thromboembolism [10, 11]. After the detorsion of tissue, ischemia may resolve, resulting in tissue recirculation and reperfusion. However, this procedure also has adverse effects, including reperfusion injury [12]. Ischemia followed by reperfusion of the tissue releases reactive oxygen species (ROS), which, together with its associated products, damage cell membranes and worsen tissue functions. There have also been studies reporting the benefits of antioxidant therapy for ischemia-reperfusion injury in different tissue types. Hartmann et al. reported that pretreatment with glutamine which, is well known as an antioxidant, is useful in protection against oxidative damage in the intestine and liver in an experimental study [13].

Metformin, a biguanide group agent, is used as an insulin sensitizer for the treatment of diabetes. In a few reported studies, it has been speculated that metformin has antioxidant and anti-inflammatory effects [14]. It enhances the use of glucose in peripheral tissues and increases cyclic adenosine mono phosphate kinase (AMPK) which plays a major regulatory role in the balance of cellular energy by switching cells from the anabolic state to the catabolic state [15]. It has been reported that metformin has an ability to decrease inflammation by reducing ROS, by decreasing the activity of mitochondrial complex I [16]. Besides, its anti-inflammatory effects rely on inhibition of the activation of NF-κB and activation of AMPK [17]. 

In this study, we aimed to evaluate the efficacy of metformin therapy in addition to detorsion for preserving ovarian reserve and ovarian structure. 

## 2. Materials and methods

This study was performed at a tertiary medical centre named Bağcılar Training and Research Hospital Experimental Laboratory, İstanbul after the approval by the ethics committee of the animal studies of the same institution. A power analysis was performed to calculate the minimum sample size required for animal studies, considering anti-Müllerian hormone (AMH) results, (alpha error: 0.05; beta: 0.8) suggested that ≥12 ovaries were required for each study group. Because 10% of study animals are lost during procedures, we included 16 ovaries per surgery group. We planned to include 8 rats in each group, and 24 female, nonpregnant, Wistar Hannover rats (reproductive age, 8 weeks; weight, 180–260 g; with 2 consecutive 4-day-long oestrous cycles proven with daily vaginal smears) were finally included in the study. All rats were kept in standard laboratory conditions (minimum cage area, 350 cm2; minimum cage height, 14 cm; fed *ad libitum* with rat pellets; mean temperature, 21 °C; 12-h day-night period; mean humidity, 50%). 

**Figure 1:**
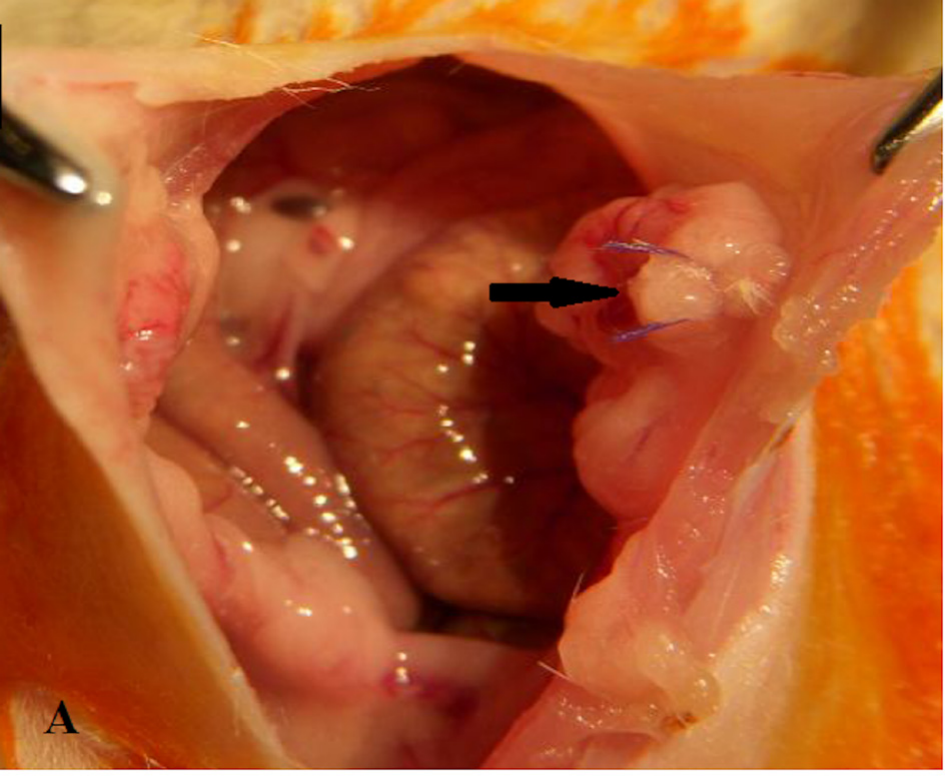
The black arrow shows exposed ovary and uterine horn. The adnexa is rotated 360° clockwise, and then sutured to the abdominal wall.

Three groups (n = 8 for each group) were generated through a computer-based randomization. Group 1 was the control group, which underwent laparotomy only; group 2 was the detorsion only group; and group 3 was the detorsion + metformin group. General anesthesia was induced with 50 mg/kg intramuscular ketamine hydrochloride (10% Ketasol; Richter Pharma AG, Weis, Austria) and 5 mg/kg intramuscular xylazine hydrochloride (2% Rompun; Bayer HealthCare LCC, Shawnee, KS, USA) in sterile conditions. The rat was laid on the operation table in the supine position. Before the surgeries, 1 mL of blood samples were drawn from the jugular vein for rat AMH analysis. The operation field was shaved, and 10% povidone-iodine solution was used for antisepsis. The uterine horns and both ovaries were exposed after making a 3-cm midline abdominal incision. The control group underwent laparotomy only with no extra intervention; the detorsion only and metformin and detorsion groups both had the ovaries rotated 360° and fixed to the abdominal side wall with 4–0 vicryl (Figure 1). The peritoneum was closed with 4–0 polyglycolic acid sutures, and 3–0 silk was used for skin closure. 

**Figure 2:**
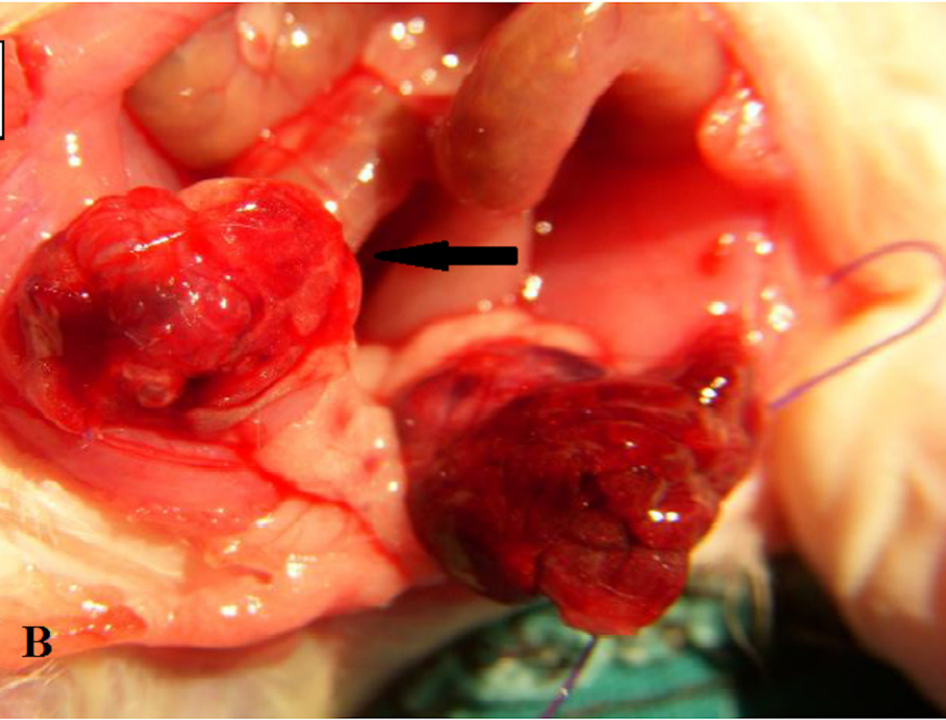
The black arrow shows torsioned ovary after 3h ischemia period.

After each operation, the operation field was sterilized, and the other procedure was started after 15 min. A 3-h-long torsion and detorsion period were selected considering the data from previous study demonstrating that histologic changes in the ovaries can occur after 3 h of continuous ischemia [18]. After a 3-h ischemia period, second operations were performed with the induction of the same anaesthesia (Figure 2). The ovaries in the detorsion only and metformin and detorsion groups were released from the abdominal side wall and detorsioned, and the abdominal incision was closed. After the operations, 1 rat in each group died of intolerance of the second dose of anaesthesia. The study continued with 7 rats in each group. Other than the other groups, only metformin+detorsion group received 50 mg/kg/day metformin (Glucophage 500 mg tablet; Merc İlaç Sanayi A.Ş, İstanbul, Turkey) for 14 days by gavage.

After 3 oestrous cycles, which correspond to three menstrual cycles in humans, 1 mL of blood samples were drawn from the jugular vein of the each rat for a second AMH analysis. Laparotomy was performed in each rat, and bilateral oophorectomies were performed for histopathological analysis. Each rat was euthanized by means of cervical dislocation after the operations. 

### 2.1. Histopathology 

**Figure 3:**
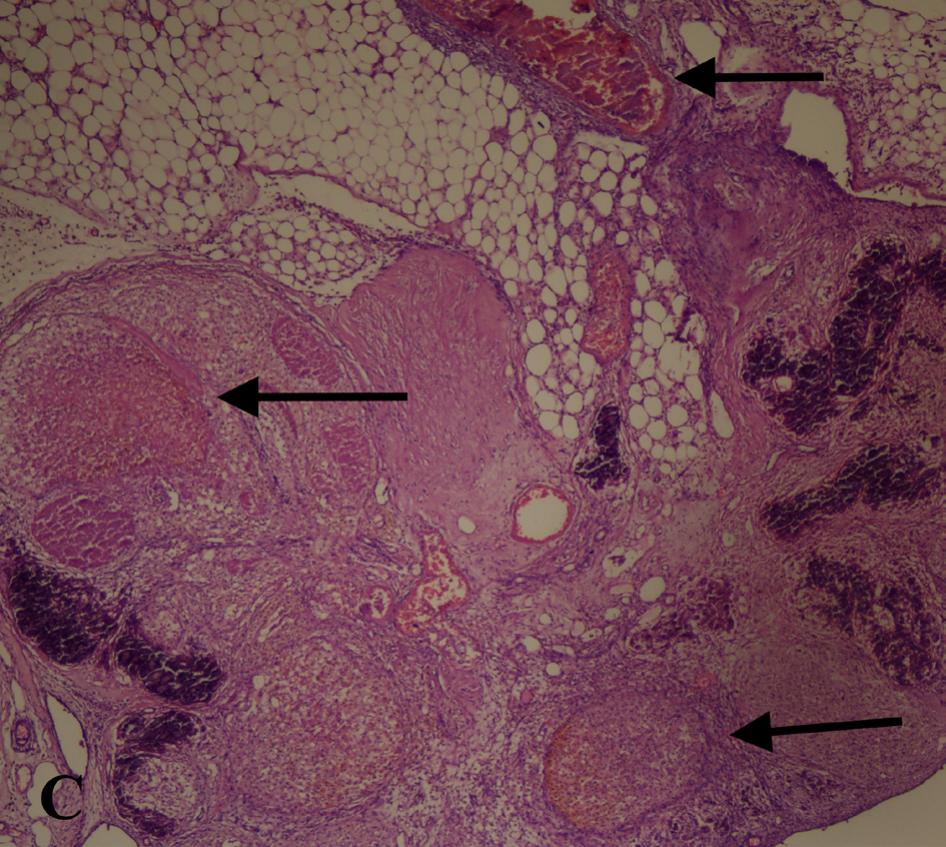
The black arrows show haemorrhage area with red blood cells. H and Ex200

**Figure 4:**
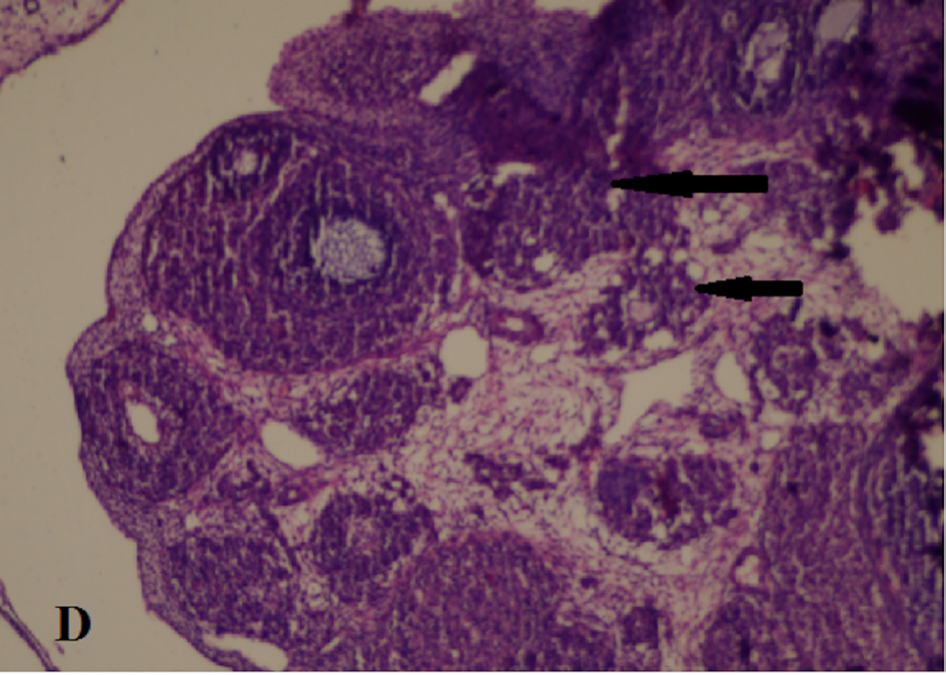
The black arrows show degenerated granulosa cells. H and Ex200

The excised ovaries were kept in 10% formalin solution and evaluated after 24 h by a pathologist from Bakırköy Dr Sadi Konuk Training and Research Hospital who was blinded to the study groups. The tissue samples were embedded in paraffin blocks, cut into 4-μm slices, and prepared for haematoxylin and eosin staining. Follicle counting was performed according to a study by Ozler et al. [19]. At least 5 microscopic areas were evaluated with light microscopy (Nikon Eclipse 80i AS Amstelveen, The Netherlands). The follicles were divided into 4 groups according to diameter: primordial (<20 μm), preantral (20–220 μm), small antral (221–310 μm), and large antral (311–370 μm). Atretic follicles were defined according to the study by Osman et al. [20]. The ovarian damage score was evaluated on the basis of the following parameters: follicle cell degeneration, vascular congestion, haemorrhage, and inflammation for both ovaries (0: none, 1: mild, 2: moderate, 3: severe) (Figure 3,4). 

### 2.2. AMH analysis

All blood samples were centrifuged for 10 min at 4000 rpm to obtain serum samples. The samples were kept at –80 °C in Eppendorf tubes until analysis. Serum AMH levels were analysed with an automatic ELISA kit (Omentin ELISA kit; Hangzhou Eastbiopharm Co., Hangzhou, China). 

### 2.3. Statistical analysis

Statistical analyses were performed by using NCSS (Number Cruncher Statistical System) 2007 statistical software (Kaysville, UT, USA). Descriptive statistical analysis, such as mean, standard deviations, and one-way analysis of variance, was used for continuous data with a normal distribution. A valid chi-squared test is used for categorical variables. The Tukey test was used for post hoc analysis of normally distributed parametric data. A paired sample t-test was used to evaluate the preoperative and postoperative AMH levels. The Kruskal–Wallis test was used to compare continuous data with skewed distribution, and the Dunn test was used for post hoc analysis. The chi-square test was used for qualitative data. P values of <0.05 were considered statistically significant.

## 3. Results

The ovarian damage scores were evaluated for each rat, and there was a statistically significant difference in the follicular damage scores between the control, detorsion only, and metformin and detorsion groups (P = 0.048). There was a mildly increased damage score in the control and metformin and detorsion groups. However, a severe score was observed in the detorsion only group, which was statistically significant in comparison with the other groups. There was also a statistically significant difference in the inflammation scores among the study groups (P = 0.002). No inflammation was observed in the control and metformin and detorsion groups. However, moderate and severe scores were observed in the detorsion only group. The total damage scores were statistically different among the study groups. The total damage scores were higher in the detorsion only group than in the control and metformin and detorsion groups (P = 0.005 and P = 0.021, respectively). However, there was no statistical difference in the total damage score between the control group and the metformin and detorsion group. There was no statistically significant difference in vascular congestion and haemorrhage scores among the study groups (Table 1, 2). 

**Table 1:** Follicle cell degeneration, vascular congestion, haemorrhage, inflammation, and total damage scores between study groups.

		Control Group(n,%)		DetorsionOnly Group (n,%)		Metformin and detorsion Group (n,%)		P- value
Follicle cell degeneration	None	1	7.14%	2	14.29%	2	14.29%	0.048
	Mild	8	57.14%	5	35.71%	7	50.00%	
	Moderate	5	35.71%	2	14.29%	5	35.71%	
	Severe	0	0.00%	5	35.71%	0	0.00%	
Vascular congestion	None	1	7.14%	0	0.00%	0	0.00%	0.069
	Mild	9	64.29%	5	35.71%	5	35.71%	
	Moderate	4	28.57%	6	42.86%	9	64.29%	
	Severe	0	0.00%	3	21.43%	0	0.00%	
Haemorrhage	None	2	14.29%	4	28.57%	2	14.29%	0.075
	Mild	10	71.43%	3	21.43%	10	71.43%	
	Moderate	2	14.29%	3	21.43%	1	7.14%	
	Severe	0	0.00%	4	28.57%	1	7.14%	
Inflammation	None	12	85.71%	4	28.57%	11	78.57%	0.002
	Mild	2	14.29%	2	14.29%	3	21.43%	
	Moderate	0	0.00%	6	42.86%	0	0.00%	
	Severe	0	0.00%	2	14.29%	0	0.00%	
*Total damage score 3.64 ± 1.34 6.5 ± 3.32 4.14 ± 1.46 0.004								

Chi-square test, *One Way ANOVA

(0: none, 1: mild, 2: moderate, 3: severe).

P-value of <0.05 was considered to be statistically significant.

The study groups were also evaluated for follicle counts. There was a statistically significant difference in the numbers of preantral follicles, large antral follicles, and corpora lutea among the control, detorsion only, and metformin and detorsion groups (P = 0.0001, P = 0.041, P = 0.023, respectively) (Table 3). The preantral follicle, large antral follicle, and corpora lutea counts were lower in the detorsion only group than in the control and metformin and detorsion groups. There was no significant difference in preantral follicle, large antral follicle, and corpora lutea counts between the control group and the metformin and detorsion group (Table 4). There was also no significant difference in the counts of primordial, small antral, and atretic follicles between the study groups. There was a statistically significant difference in the preoperative vs. postoperative anti-Müllerian hormone (AMH) level changes between the control, detorsion only, and metformin and detorsion groups (P = 0.0001) (Table 5). The detorsion only group showed negatively statistically different changes in this parameter compared with the other groups (P = 0.001) (Table 6). However, there was no statistically significant difference for this parameter between the control group and the metformin and detorsion group. 

**Table 2:** Tukey HSD test P-values for the total damage scores of the study groups.

Tukey HSD test	Total damage score
Control group/detorsion only group	0.005
Control group/Metformin and detorsion group	0.825
Detorsion only group/Metformin and detorsion group	0.021

**Table 3:** Primordial, preantral, small antral, large antral and atretic follicle counts, and corpora lutea counts of the study groups.

		Controlgroup	Detorsiononly group	Metformin anddetorsion group	P-value
Primordial follicle	Mean ± SD	4 ± 2.18	2.86 ± 4.22	4 ± 2.6	0.112
		(2–5)	(0–4)	(1.75–5.25)	
Preantral follicle	Mean ± SD	3.57 ± 1.65	0.79 ± 0.89	3 ± 2.04	0.0001	(Range)	(2–5)
	(0–2)	(1–3.5)			
Small antral follicle	Mean ± SD	4 ± 2.48	2.57 ± 2.44	3.21 ± 1.76	0.291
	(Range)	(3–4)	(0–4)	(1.75–5)	
Large antral follicle	Mean ± SD	10.36 ± 5.03	5.57 ± 5.17	10.14 ± 5.53	0.041
	(Range)	(7.25–14.25)	(0–9.25)	(4–14.25)	
Corpora lutea	Mean ± SD	6.36 ± 3.59	2.93 ± 3.71	5.93 ± 3.77	0.023
	(Range)	(4.75–9)	(0–5)	(2.8–10)	
Atretic follicle	Mean ± SD	23.79 ± 17.5	26.36 ± 14.14	23.57 ± 9.94	0.502
	(Range)	(15–25.75)	(14.75–39.5)	(13–31)	

Kruskal–Wallis test SD: Standard deviation P-value of <0.05 was considered to be statistically significant.

**Table 4:** Dunn’s test P-values for the preantral follicle, large antral follicle, and corpora lutea counts of the study groups.

Dunn’s test	Preantralfollicle	Large antralfollicle	Corporalutea
Control group/detorsion only group	0.0001	0.022	0.008
Control group/Metformin and detorsion group	0.315	0.818	0.889
Detorsion only group/Metformin and detorsion group	0.001	0.04	0.037

**Table 5:** Preoperative and postoperative serum AMH results of the study groups.

	Control group	Detorsiononly group	Metformin anddetorsion group	P- value*
Preoperative AMH	4.34 ± 1.43	5.87 ± 1.02	3.65 ± 1.42	0.0001
Postoperative AMH	3.22 ± 1.39	1.78 ± 1.82	2.54 ± 1.18	0.048
P-value ¶	0.0001	0.0001	0.0001	
Pre-Postoperative AMH alteration	1.12 ± 0.24	4.09 ± 1.14	1.12 ± 0.69	0.0001

AMH (anti-Müllerian hormone) ¶ paired sample t test *Kruskal–Wallis test Note: Values are expressed as mean ± SD

**Table 6:** Tukey HSD test P-values for the preoperative, postoperative serum AMH results, and pre-postoperative AMH alteration of the study groups.

Tukey HSD test	Preoperative AMH	Postoperative AMH	Pre-Postoperative AMH alteration
Control group/detorsion only group	0.01	0.037	0.0001
Control group/Metformin and detorsion group	0.349	0.450	0.998
Detorsion only group/Metformin and detorsion group	0.001	0.380	0.0001

AMH (anti-Müllerian hormone)

## 4. Discussion

In present study, our aim was to evaluate the efficacy of metformin considering its antioxidant and antiinflammatory benefits in addition to detorsion for preserving ovarian reserve and ovarian structure and we revealed that Metformin and detorsion treatment may be effective in protecting the ovarian reserve after ovarian torsion.

Excision of the adnexa is the traditional approach for treating ovarian torsion. However, recent studies do not recommend this treatment approach, considering the importance of the ovary for women of reproductive age [21,22]. Moreover, there is still some concern about leaving necrotic tissue in situ and complications such as infection, increased risk of malignancy, and systemic problems such as pulmonary embolism or thrombosis in other organs [23]. In our study, our main goal was to determine the ovarian reserve after ovarian detorsion and we did not observe any signs of pelvic infection. 

Ischemia-reperfusion injury is generally explained by the hypothesis that there is an accumulation of neutrophils and thrombocytes due to the activated complement and other inflammatory components around the inflammation site. This aggregation of inflammatory cells enhances the production of ROS. In addition, glycolysis, increased lactic acid concentration, and intracellular Ca accumulation result in decreased intracellular pH and acidosis. This results in increased intracytoplasmic lysozyme enzymes causing damage to proteins and the cell membrane [24]. Enzymes such as glutathione peroxidase and catalase play a protective role against cellular ROS. Moreover, cysteine, glutathione, ceruloplasmin, and vitamins A/C/E also act as intracellular and extracellular antioxidants and protect the cell structure from ROS [25]. The balance between oxidants and antioxidants is lost in ischemia-reperfusion injury. In this case, enzymes such as lipid peroxidase, superoxide dismutase, inducible nitric oxide synthase, and myeloperoxidase levels increase [26,27]. In a study by Bostancı et al. [28], granulocyte colony-stimulating factor (G-CSF), a glycoprotein commonly used to treat neutropenia by mobilizing bone marrow-derived hematopoietic cells into peripheral blood, was used in an experimental model of ischemia-reperfusion injury. The authors administered intraperitoneal injections of G-CSF (100 IU/kg) and evaluated the mean total oxidant status (TOS), oxidative stress index (OSI), and the total histopathological scores of rats with ischemia-reperfusion injury. G-CSF administration decreased the mean TOS and OSI levels significantly when compared with the controls. Moreover, there was a decrease in total histopathological scores for rats conservatively treated with G-CSF compared with the control groups. Bakacak et al. [29] used platelet-rich plasma, which is clinically used to promote wound healing, in an experimental ischemia-reperfusion injury model. They found that the TOS, OSI, and total ovarian histopathological scores were higher in the nontreated group than in the group treated with 0.5 mL platelet-rich plasma. Halici et al. [27] evaluated the long-acting calcium channel blocker amlodipine in an experimental model of ischemia-reperfusion injury. They administered 3 and 5 mg/kg doses of amlodipine, and concluded that amlodipine is effective in preventing ovarian damage. Kumtepe et al. [30] studied the angiotensin 2 type 1 receptor blocker telmisartan, which is used as an antihypertensive agent in daily practice, in an experimental ischemia-reperfusion model. Telmisartan, at doses of 10 and 20 mg/kg, reduced ROS, inducible nitric oxide synthase activity, and myeloperoxidase levels compared with no treatment and all other tested drug doses. In the line with our study, Sayan et al. evaluated the effect of metformin on ovarian ischemia-reperfusion injury in an experimental torsion/detorsion rat model. They used the glutathione-to-oxidized-glutathione ratio, malondialdehyde (MDA), and caspase-3 activation to determine tissue damage in addition to ovarian histopathology. According to their results metformin was effective in regards to attenuating ovarian ischemia-reperfusion injury [31]. All of the studies mentioned above are based on protecting the cell from these ROS [32]. In our study, we also tried to evaluate antioxidant and antiinflammatory effect of metformin after ovarian detorsion. Previous ischemia-reperfusion studies performed with metformin in different tissues have shown that metformin protects tissues by reducing lipid peroxidase, MDA, ROS, and inflammatory cytokines [33,34]. Furthermore, it has been reported that metformin relieves the inflammatory responses after cardiac ischemia by activating adenosine monophosphate-activated protein kinase (AMPK). AMPK agonists also inhibit inflammatory factors such as tumour necrosis factor-α, interleukin-6, and cyclooxygenase-2 [35,36]. In our study, we found that the follicular damage scores and inflammatory damage scores were significantly better in the group that received metformin than in the group that received detorsion only. We may postulate that this effect results from the antioxidant and antiinflammatory effects of the drug.

The status of the ovarian reserve can be determined on the basis of the antral follicular counts on ultrasound, serum day 3 follicle-stimulating hormone and oestradiol levels, clomiphene citrate challenge test, and anti-Müllerian hormone (AMH) levels [37]. In addition to the study by Sayan et al. [31], in our study, we evaluated the status of ovarian reserve by measuring AMH, which is released from preantral and small antral follicles. We found that the preoperative to postoperative decrease was greater in the detorsion only group than in the control and metformin and detorsion groups. 

As a limitation of our study, the duration of postoperative period may not be enough to determine ovarian damage. In addition, reperfusion injury may be evaluated with oxidative stress markers in serum or tissue samples. These limitations may be considered in future studies.

## Informed Consent

None (experimental animal study)
